# Curcumin alleviates diabetic encephalopathy by attenuating cerebral microvascular ferroptosis: involvement of KEAP1/Nrf2 signalling

**DOI:** 10.3389/fphar.2026.1914347

**Published:** 2026-09-21

**Authors:** Zhaoqi Zhang, Dongxian Wang, Xingru Zhao, Shang Gao, Fangwei Xu, Xiaowen Yv, Zhenyuan Jiang, Tong Wu, Moji Wangchen, Wei Liu, Xin Li

**Affiliations:** 1 The First Clinical Medical College, Shandong University of Traditional Chinese Medicine, Jinan, China; 2 Department of Neurology, Affiliated Hospital of Shandong University of Traditional Chinese Medicine, Jinan, China; 3 Military Retirement Clinic, Jinan Municipal Government Hospital, Jinan, Shandong, China; 4 Department of Imaging, Affiliated Hospital of Shandong University of Traditional Chinese Medicine, Jinan, China; 5 Department of Neurology, The Second Affiliated Hospital of Shandong University of Traditional Chinese Medicine, Jinan, China

**Keywords:** cerebral microvessels, curcumin, diabetic encephalopathy, ferroptosis, KEAP1, Nrf2

## Abstract

**Background:**

Cerebral microvascular injury and ferroptosis are increasingly implicated in diabetic encephalopathy (DE). This study investigated whether curcumin (Cur) alleviates DE by attenuating cerebral microvascular ferroptosis and examined the involvement of KEAP1/Nrf2-related signalling.

**Methods:**

Diabetic rats were treated with Cur for 8 weeks, with metformin as a positive control. Metabolic parameters, cognitive function, hippocampal histopathology, cerebral microvascular tight-junction proteins, oxidative stress, and ferroptosis-related markers were evaluated. Brain untargeted metabolomics and exploratory network analysis were integrated with molecular experiments to generate and experimentally evaluate mechanistic hypotheses. An HGHF-induced bEnd.3 cell model, together with erastin, ferrostatin-1, and ML385 interventions, was used for *in vitro* validation.

**Results:**

Cur improved metabolic abnormalities, cognitive impairment, and hippocampal injury in diabetic rats. Untargeted metabolomics revealed ferroptosis-related metabolic alterations, including changes in glutathione-, taurine-, and polyunsaturated fatty acid-related metabolism. Cur reduced oxidative stress, restored tight-junction protein expression, and attenuated ferroptosis-related changes in cerebral microvessels. Experimental analyses further showed coordinated regulation of KEAP1, NRF2, HO-1, GCLC, GPX4, ACSL4, and NOX4 *in vivo*. In HGHF-treated bEnd.3 cells, Cur reduced lipid peroxidation and Fe^2+^ accumulation, improved antioxidant capacity, attenuated mitochondrial injury, and restored tight-junction protein expression. Erastin aggravated, whereas ferrostatin-1 attenuated, HGHF-induced ferroptosis-related injury, and ML385 partially attenuated the protective effects of Cur.

**Conclusion:**

Cur alleviated DE-related metabolic, cognitive, and hippocampal abnormalities and attenuated ferroptosis-associated cerebral microvascular injury. KEAP1/Nrf2-related signalling contributed to these protective effects. These findings link Cur-mediated neuroprotection to the regulation of cerebral microvascular ferroptosis and support its further investigation in diabetes-associated cognitive impairment.

## 1 Introduction

Diabetic encephalopathy (DE) is a chronic central nervous system complication caused by diabetes, primarily characterised by progressive cognitive decline and abnormalities in brain structure and function (McCrimmon et al., 2012). With rising diabetes prevalence and an aging population, DE has become an increasingly prominent public health issue (Moheet et al., 2015). Studies have shown that DE is mediated by multiple mechanisms, including hyperglycaemia-related metabolic disturbances, oxidative stress, mitochondrial dysfunction, neuroinflammation, microvascular damage, and disruption of the blood-brain barrier (BBB) (Luo et al., 2024). Currently, clinical management primarily focuses on controlling blood glucose levels and managing risk factors; there is still no specific treatment for DE (Areosa Sastre et al., 2017).

Cerebral microvascular injury has emerged as an important mechanism in the development of DE (van Sloten et al., 2020). Brain microvascular endothelial cells (BMECs), the principal cellular component of the BBB, are essential for maintaining central nervous system homeostasis (Wątroba et al., 2023). Under diabetic conditions, metabolic stress impairs endothelial function and BBB integrity, thereby contributing to neuronal injury and cognitive decline (He et al., 2025). Accordingly, microvascular dysfunction is now recognised as a key pathological feature of DE.

Ferroptosis has recently been recognised as an important mechanism linking diabetic metabolic stress to neurovascular injury in DE (Ma et al., 2025). As an iron-dependent form of regulated cell death driven by lipid peroxidation, ferroptosis is characterised by glutathione depletion, glutathione peroxidase 4 (GPX4) inactivation, and oxidative membrane damage (Liu et al., 2024). Further evidence indicates that ferroptosis impairs BBB function and disrupts BMEC homeostasis, highlighting its pathological relevance in diabetic brain injury (Shi et al., 2023; Zhao et al., 2023). Kelch-like ECH-associated protein 1 (KEAP1)/nuclear factor erythroid 2-related factor 2 (Nrf2) signalling is one of the most important endogenous protective pathways against ferroptotic stress, as Nrf2 activation promotes the transcription of antioxidant- and glutathione-related genes, thereby restraining lipid peroxidation and redox imbalance (Cheng et al., 2024b). Conversely, dysregulation of this pathway has been closely associated with DE and other diabetes-related injuries, suggesting that targeting KEAP1/Nrf2 signalling may be an effective strategy for alleviating ferroptosis-related neurovascular damage in DE (Ebrahimi et al., 2025).

Curcumin (Cur), a major polyphenolic constituent of *Curcuma longa* L., has been extensively investigated for its antioxidant, anti-inflammatory, neuroprotective, and vasculoprotective properties, with accumulating evidence suggesting beneficial effects against diabetes-associated brain injury (Miao et al., 2021; Tian et al., 2025). Notably, Cur can activate antioxidant signalling by promoting Nrf2 stabilisation and nuclear translocation and enhancing the expression of downstream cytoprotective genes (Park et al., 2021). However, Cur has also been recognised as a pan-assay interference compound (PAINS), which raises concerns regarding promiscuous, nonspecific, or assay-dependent effects, particularly when mechanistic interpretation relies predominantly on computational prediction or isolated *in vitro* assays (Nelson et al., 2017; Bolz et al., 2021; Magalhães et al., 2021). Accordingly, computational findings involving Cur require cautious interpretation and experimental validation using complementary biological approaches. The KEAP1/Nrf2 axis may also provide a mechanistic link between diabetic cerebral microvascular injury and ferroptosis. Activation of Nrf2 has been shown to preserve tight-junction proteins and reduce blood–brain barrier permeability in type 2 diabetic mice and human brain microvascular endothelial cells exposed to hyperglycaemic and inflammatory stress (Zhao et al., 2019). Moreover, ferroptosis of brain microvascular endothelial cells, characterised by impaired GSH/GPX4 defence and enhanced lipid peroxidation, contributes to tight-junction disruption and blood–brain barrier injury (Liu et al., 2023). These findings provide a rationale for investigating KEAP1/Nrf2-related signalling in Cur-mediated protection against cerebral microvascular ferroptosis. However, whether this signalling axis contributes to Cur-mediated protection against cerebral microvascular ferroptosis in DE remains unclear, and previous studies have largely focused on the general antioxidant and anti-inflammatory effects of Cur without integrating metabolic alterations with endothelial ferroptosis-related mechanisms. Therefore, we combined brain non-targeted metabolomics, network analysis, cerebral microvessel analysis, and an HGHF-induced bEnd.3 cell model to characterise ferroptosis-associated metabolic and molecular changes and to evaluate the involvement of KEAP1/Nrf2-related signalling. This integrated approach extends previous findings by linking Cur-induced metabolic regulation with cerebral microvascular ferroptosis and pathway-level experimental validation in DE.

## 2 Materials and methods

### 2.1 Animals and ethical approval

Specific pathogen-free male Sprague–Dawley rats (5 weeks old, 160–180 g) were purchased from Beijing Vital River Laboratory Animal Technology Co., Ltd. (Beijing, China; licence No. SCXK [Jing] 2021-0006). Rats were housed under standard conditions (22 °C ± 2 °C, 55% ± 5% relative humidity, and a 12-h light/dark cycle) with free access to food and water and were acclimatised for 1 week. Sample sizes were determined with reference to previous studies using high-fat diet/STZ-induced diabetic rats and preliminary experimental variability. At least six biological replicates were included in the principal animal experiments, and the exact sample size for each analysis is provided in the corresponding figure legend. All procedures were approved by the Animal Ethics Committee of the Affiliated Hospital of Shandong University of Traditional Chinese Medicine (approval No. SDUTCM20230314001) and complied with the Guide for the Care and Use of Laboratory Animals and the ARRIVE guidelines.

### 2.2 Reagents

Cur (purity 98.63%), streptozotocin (STZ), erastin, ferrostatin-1 (Fer-1), and ML385 were purchased from MedChemExpress. Metformin (Met) was obtained from Merck Pharmaceutical, and palmitic acid (PA) was obtained from Sigma-Aldrich. Cell culture reagents were purchased from Gibco or Servicebio. Primary antibodies against ZO-1, Occludin, Claudin-5, ACSL4, GPX4, NRF2, HO-1, GCLC, KEAP1, NOX4, OPA1, MFN1, MFN2, and DRP1 were obtained from ABclonal Technology. The β-actin antibody was obtained from Servicebio.

### 2.3 Safety assessment, diabetic model establishment, and drug administration

Cur was suspended in corn oil for oral administration. The oral doses of 200 mg/kg (approximately 0.543 mmol/kg) and 400 mg/kg (approximately 1.086 mmol/kg) were selected with reference to previous experimental studies in diabetic rats, including studies employing the same dose range, and were used to examine biological responses across two experimental dose levels (Faheem and El Askary, 2017; Febriza et al., 2024). These doses were intended to characterise pharmacological responses within the established rat model rather than to imply direct correspondence with clinical dosing. For the safety assessment, healthy rats were assigned to the control (Con), CurL (200 mg/kg), or CurH (400 mg/kg) group. The randomisation sequence was generated using computer-generated random numbers by an independent investigator who was not involved in treatment or outcome assessment. Cur was administered by oral gavage once daily for 8 weeks, whereas the Con group received an equal volume of corn oil. Body weight, fasting blood glucose, and serum ALT, AST, TG, and TC levels were measured. Type 2 diabetes was induced using a high-fat diet (HFD) combined with low-dose STZ (Srinivasan et al., 2005; Miao et al., 2015). The HFD comprised 70% basal chow, 16% sucrose, 12% lard, 1% cholesterol, and 1% sodium cholate. After 4 weeks of HFD feeding, rats received STZ (30 mg/kg, intraperitoneally), whereas the Con group received citrate buffer. Rats with random blood glucose ≥16.7 mmol/L on two consecutive measurements were considered diabetic. Eligible diabetic rats were assigned to the Mod, Mod-CurL, Mod-CurH, or Mod-Met group using a second computer-generated randomisation sequence prepared by the independent investigator. Cur at 200 or 400 mg/kg or Met at 200 mg/kg was administered orally once daily for 8 weeks. The Con and Mod groups received an equal volume of vehicle.

### 2.4 Metabolic and biochemical assessments

Body weight was recorded weekly. Fasting blood glucose was measured after overnight fasting. For the oral glucose tolerance test, glucose (2.0 g/kg) was administered after 12 h of fasting, and blood glucose was measured at 0, 30, 60, 90, and 120 min. The area under the curve was calculated. At study termination, serum ALT, AST, TG, TC, and fasting insulin levels were measured using commercial kits. HOMA-IR was calculated as fasting blood glucose (mmol/L) × fasting insulin (mIU/L)/22.5, and the TyG index was calculated as ln [fasting TG (mg/dL) × fasting blood glucose (mg/dL)/2] (Rad et al., 2024). Samples were coded before measurement, and the investigators performing the assays were blinded to group allocation.

### 2.5 Morris water maze test

Spatial learning and memory were assessed using the Morris water maze. Rats underwent five consecutive days of acquisition training, with a maximum trial duration of 60 s, followed by a 60-s probe trial on day 6. Escape latency and swimming distance were recorded during training. Time and distance in the target quadrant, platform crossings, and swimming trajectories were recorded during the probe trial. The testing order was randomised daily, and behavioural testing and data analysis were performed by investigators blinded to group allocation.

### 2.6 Histopathological examination

Brain tissues were fixed in 4% paraformaldehyde, paraffin-embedded, sectioned at 4 μm, and subjected to haematoxylin and eosin staining. Hippocampal pathological changes were examined using a light microscope. Sections were coded by an independent investigator, and image acquisition and evaluation were performed under blinded conditions.

### 2.7 Non-targeted metabolomics analysis

Brain tissues from the Con, Mod, and Cur-treated groups were analysed by non-targeted metabolomics, with six biological replicates per group. Samples were randomly selected, coded, and analysed in a randomised injection order. After extraction and low-temperature centrifugation, supernatants were subjected to LC–MS/MS analysis. Pooled quality-control samples were prepared by combining equal aliquots from all samples and were inserted regularly throughout the analytical sequence. Analytical stability was assessed from QC clustering in principal component analysis and the relative standard deviation of detected features. Features with an RSD >30% in QC samples were excluded. Raw data were processed for peak detection, retention-time alignment, peak-area integration, missing-value imputation, normalisation, and duplicate removal. Normalised intensities were log_2_-transformed for statistical analysis. Pareto-scaled and unit-variance-scaled data were used for PCA and partial least-squares discriminant analysis, respectively. Fold changes were calculated from normalised peak intensities, and P values were adjusted using the Benjamini–Hochberg method. Differential metabolites were defined by a fold change ≥1.5 or ≤1/1.5 and an adjusted P value < 0.05. Reversed metabolites were defined as metabolites significantly altered in the Mod versus Con comparison and significantly changed in the opposite direction in the Cur versus Mod comparison. Representative ferroptosis-associated metabolites were further evaluated using log_2_ fold changes, adjusted P values, and individual log_2_-normalised intensities. KEGG pathway classification and enrichment analyses were performed for differential and reversed metabolites. Pathways with an adjusted P value < 0.05 were considered significantly enriched.

### 2.8 Isolation of cerebral microvessels

Cerebral microvessel-enriched fractions were isolated from the rat cortex using a previously described method (Lee et al., 2019). After the rats were deeply anaesthetised, their brains were rapidly removed and placed on ice. The cerebral cortex was then dissected and the meninges, white matter and large surface vessels were removed. The cortical tissue was then homogenised in ice-cold PBS, mixed with a dextran solution and centrifuged at 5,400  *g* for 15 min at 4 °C. The resulting pellet was resuspended and sequentially filtered through 100- and 40-μm nylon meshes. The retained fraction was washed with ice-cold buffer solution and collected for subsequent protein analyses.

### 2.9 Network analysis and enrichment analysis

Predicted Cur-associated proteins were retrieved from TCMSP, HERB, PharmMapper, and SwissTargetPrediction, with the latter used as a ligand-similarity-based target-prediction resource (Daina et al., 2019). DE-related genes/proteins were collected from GeneCards, DisGeNET, and OMIM, and ferroptosis-related genes/proteins were obtained from GeneCards. GeneCards relevance-score thresholds were >10 for DE and >5 for ferroptosis. All resources were searched on 25 July 2025. These databases and prediction tools were used solely for exploratory candidate generation. Because different resources may share underlying literature, interaction records, or computationally inferred annotations, recurrence across databases was not regarded as independent replication or as evidence of pharmacological activity. After duplicate removal, descriptive overlap among predicted Cur-associated proteins, DE-related genes/proteins, and ferroptosis-related genes/proteins was examined. Cytoscape version 3.10.1 was used to visualise the resulting network, and protein–protein interaction information was obtained from STRING version 11.5. GO and KEGG enrichment analyses were performed using the clusterProfiler and org.Hs.eg.db packages in R version 4.5.0. P values were adjusted using the Benjamini–Hochberg method, and terms with an adjusted P value < 0.05 were retained for descriptive interpretation.

### 2.10 Cell culture and HGHF model

Mouse brain microvascular endothelial bEnd.3 cells were maintained in DMEM containing 10% fetal bovine serum and 1% penicillin–streptomycin at 37 °C with 5% CO_2_. Cells between passages 5 and 25 were used. To establish the high-glucose/high-fat (HGHF) injury model, cells were exposed to high glucose (25 mmol/L) combined with PA at 0–600 μmol/L for 12–48 h. Control cells were cultured in medium containing 5.5 mmol/L glucose. The final modelling conditions and Cur concentrations were selected according to preliminary CCK-8 screening. Erastin, Fer-1, and ML385 were used at 10, 1, and 10 μmol/L, respectively (Huo et al., 2016; Li et al., 2021; Xu et al., 2023; Wang et al., 2024). Cells were assigned to the control, HGHF, HGHF-erastin, HGHF-Fer-1, HGHF-CurL, HGHF-CurH, HGHF-ML385, or HGHF-CurH-ML385 group using computer-generated random numbers. Culture plates were coded before treatment, and outcome assessment was performed under blinded conditions.

### 2.11 Cell viability assay and biochemical assays

Cell viability was assessed using the CCK-8 assay, with absorbance measured at 450 nm. Intracellular ROS levels were assessed using DCFH-DA fluorescence and quantified by flow cytometry. Cellular MDA, reduced GSH, and Fe^2+^ levels were determined using commercial assay kits according to the manufacturers’ instructions. MDA and GSH levels were normalised to total protein concentration. Image and plate-based measurements were analysed by investigators blinded to treatment allocation.

### 2.12 Immunofluorescence (IF) staining

bEnd.3 cells were seeded on sterile coverslips and treated as indicated. After treatment, cells were fixed with 4% paraformaldehyde for 15 min at room temperature, permeabilised with 0.1% Triton X-100 in PBS for 20 min, and blocked with 5% bovine serum albumin (BSA) for 1 h at room temperature. The cells were then incubated overnight at 4 °C with primary antibodies against Occludin (1:200), Claudin-5 (1:1000), and ZO-1 (1:100) diluted in 0.2% BSA-PBS. After washing with PBS, cells were incubated with Alexa Fluor 488-conjugated goat anti-rabbit IgG (H + L) secondary antibody (SparkJade) for 1 h at room temperature in the dark. Nuclei were counterstained with DAPI for 5 min. Fluorescence images were captured using a fluorescence microscope, and fluorescence intensity was quantified using ImageJ software.

### 2.13 Transmission electron microscopy

After treatment, bEnd.3 cells were fixed in 2.5% glutaraldehyde at 4 °C for 12 h and post-fixed in 2% osmium tetroxide for 1 h. Samples were dehydrated in a graded ethanol series, rinsed with 100% acetone for 20 min, and embedded in epoxy resin. Ultrathin sections were then stained with uranyl acetate and lead citrate and observed under a transmission electron microscope to evaluate mitochondrial ultrastructural damage.

### 2.14 Quantitative real-time PCR

Total RNA was extracted from cerebral microvessel-enriched fractions or bEnd.3 cells and reverse-transcribed into cDNA. Quantitative real-time PCR was performed using an ABI 7500 system. Relative expression was calculated using the 2^−ΔΔCt method with β-actin as the reference gene. For rat cerebral microvessels, *ACSL4*, *GPX4*, *KEAP1*, *NFE2L2*, *HMOX1*, *GCLC*, and *NOX4* were examined. For bEnd.3 cells, *ACSL4*, *GPX4*, *KEAP1*, and *NFE2L2* were examined. Primer sequences are provided in Table 1.

**TABLE 1:** Primer sequences used for RT-qPCR.

Species	Gene name	Forward (5′–3′)	Reverse (5′–3′)
Rat	ACSL4	TGC​TTC​TGC​TGC​CCC​ATT​GGT​C	AAC​CAC​CTT​CCT​GCC​AGT​CCT
	GPX4	TGA​AAG​TCC​CAG​CCC​AAG​G	CCACGCAGCCGTTCTTA
	KEAP1	GGGTGGGTGTTGCTGTC	GCT​TGT​TCT​GCT​GCC​TCT​T
	NFE2L2	CTG​CCA​TTA​GTC​AGT​CGC​T	ACCGTGCCTTCAGTGTG
	GCLC	CGG​AGG​AAC​GAT​GTC​TGA​GT	CTG​GGG​AAT​GAA​GTG​ATG​GT
	NOX4	CTG​GAC​AGA​AAG​ATT​CCG​AG	TTG​AAT​GAA​GGG​CAG​AAT​CTC​A
	HMOX1	CAA​TGA​CCC​GAA​AGA​ACT​GC	CTC​GTG​GAG​ACG​CTT​TAC​AT
	β-actin	CCT​CAC​TGT​CCA​CCT​TCC​A	GGTGTAAAACGCAGCTCA
Mouse	ACSL4	CTC​ACC​ATT​ATA​TTG​CTG​CCT​GT	TCT​CTT​TGC​CAT​AGC​GTT​TTT​CT
	GPX4	GTG​TAA​ATG​GGG​ACG​ATG​CC	ACC​ACG​CAG​CCG​TTC​TTA​TC
	KEAP1	GAT​GGG​CAG​GAC​CAG​TTG​AA	CCG​AGG​ACG​TAG​ATC​TTG​CC
	NFE2L2	ACC​TCT​GCT​GCA​AGT​AGC​CT	TGT​CAA​TCA​AAT​CCA​TGT​CCT​GC
	β-actin	GGC​TGT​ATT​CCC​CTC​CAT​CG	CCA​GTT​GGT​AAC​AAT​GCC​ATG​T

### 2.15 Western blot analysis

Proteins extracted from cerebral microvessel-enriched fractions or bEnd.3 cells were separated by SDS–PAGE and transferred onto PVDF membranes. Membranes were incubated with the indicated primary and HRP-conjugated secondary antibodies, and bands were detected using enhanced chemiluminescence. Band intensities were quantified using ImageJ and normalised to β-actin. Primary antibody dilutions were as follows: ACSL4, 1:3,000; GPX4, 1:4,000; OPA1, 1:5,000; DRP1, 1:4,000; MFN1 and MFN2, 1:1,500; HO-1, 1:5,000; NRF2, 1:1,500; GCLC, KEAP1, NOX4, and Occludin, 1:2,000; Claudin-5, 1:4,000; ZO-1, 1:3,000; and β-actin, 1:2,000. Densitometric analysis was performed using coded images under blinded conditions.

### 2.16 Statistical analysis

Data are presented as mean ± standard deviation and were analysed using GraphPad Prism version 8.0. The exact biological sample size is provided in each figure legend. Normality and homogeneity of variance were assessed before parametric analyses. Two-group comparisons were performed using an unpaired two-tailed Student’s t-test. Multiple-group comparisons were performed using one-way ANOVA followed by Tukey’s *post hoc* test. Body weight, OGTT, and Morris water maze acquisition data were analysed using two-way repeated-measures ANOVA followed by appropriate multiple-comparison tests. Metabolomic P values were adjusted using the Benjamini–Hochberg method. A two-sided *P* value < 0.05 or an adjusted *P* value < 0.05 was considered statistically significant.

## 3 Results

### 3.1 Cur improved metabolic disturbances in diabetic rats

As shown in Figures 1A,B, the experimental design and chemical structure of Cur are presented. In normal rats, 8 weeks of Cur administration did not significantly alter body weight, fasting blood glucose, or serum ALT, AST, TG, and TC levels (Figures 1C–H), indicating no detectable adverse changes in the measured safety-related parameters during the experimental period. In contrast, rats in the Mod group developed marked metabolic disturbances, including reduced body weight, impaired glucose tolerance, increased OGTT AUC and fasting blood glucose, elevated fasting serum insulin, higher serum TG and TC levels, and increased TyG index and HOMA-IR (Figures 1I–Q). These abnormalities were alleviated by Cur treatment, with CurH generally producing the greater improvement, while Met also improved most of these metabolic parameters.

**FIGURE 1:**
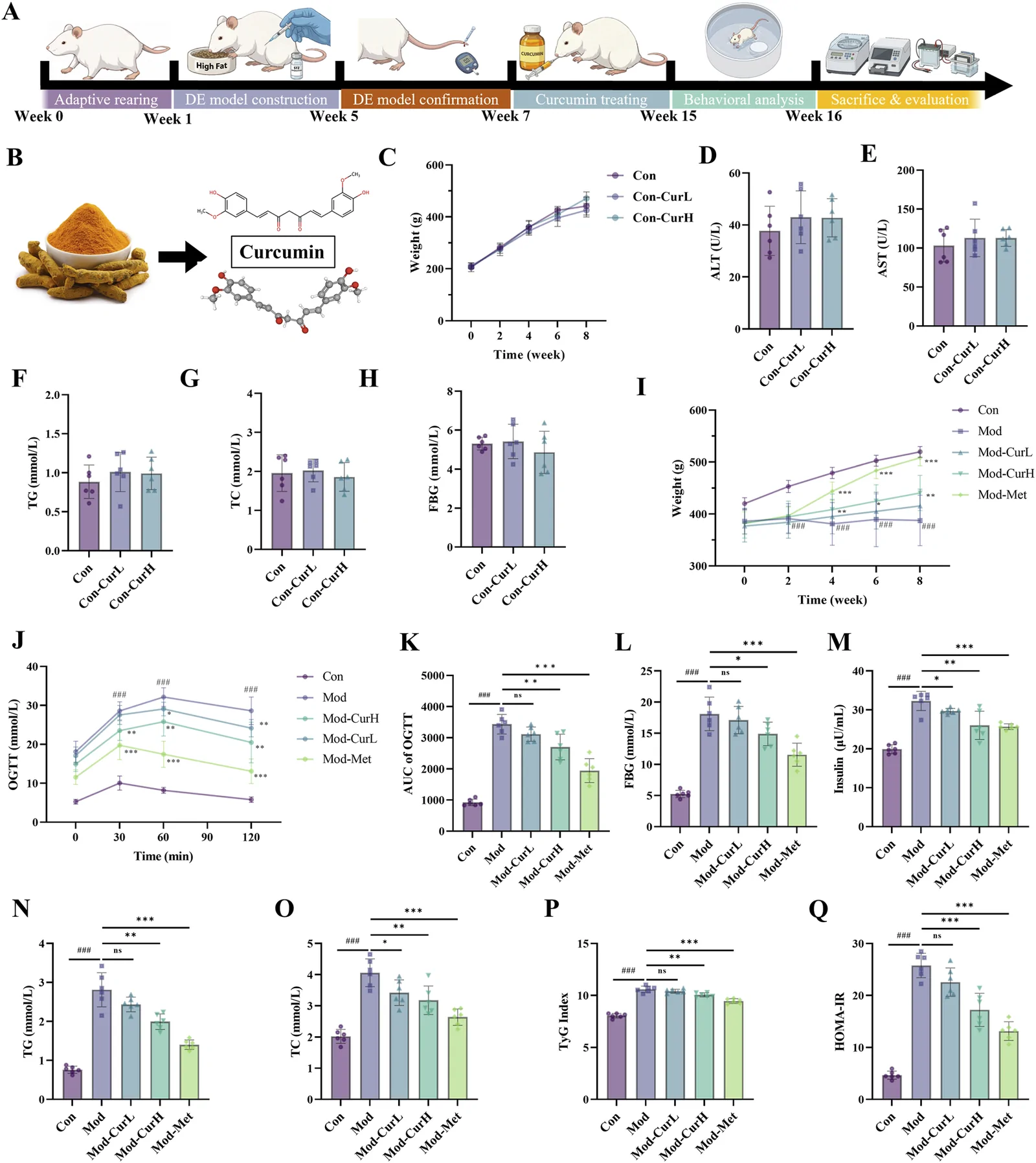
Cur ameliorated metabolic disturbances in diabetic rats. **(A)** Experimental design for diabetic encephalopathy model establishment and Cur treatment. **(B)** Chemical structure of Cur. **(C–H)** Safety evaluation of Cur in normal rats, including body weight, serum ALT, AST, TG, TC, and fasting blood glucose. **(I–Q)** Effects of Cur on metabolic parameters in diabetic rats, including body weight, OGTT, OGTT AUC, fasting blood glucose, fasting serum insulin, serum TG, serum TC, TyG index, and HOMA-IR. Data are presented as mean ± SD (n = 6). #P < 0.05, ##P < 0.01 vs. Con group; *P < 0.05, **P < 0.01 vs. Mod group. Adapted with permission from https://www.figdraw.com/#/. Used in accordance with the applicable Figdraw license and terms of use.

### 3.2 Cur improved cognitive impairment and hippocampal histopathological injury in diabetic rats

In the probe trial, rats in the Mod group displayed more disorganised search patterns and less exploration around the former platform location than those in the Con group (Figure 2A). During the acquisition phase, escape latency and swimming distance were both increased in the Mod group (Figures 2B,C). In the probe trial, the time spent in the target quadrant, swimming distance in the target quadrant, and platform crossing number were all reduced in the Mod group (Figures 2D–F). These behavioural deficits were ameliorated by Cur treatment, with CurH showing the stronger effect, while Met exhibited a similar overall tendency. H&E staining further revealed obvious hippocampal histopathological injury in the Mod group, which was alleviated after Cur treatment (Figure 2G).

**FIGURE 2:**
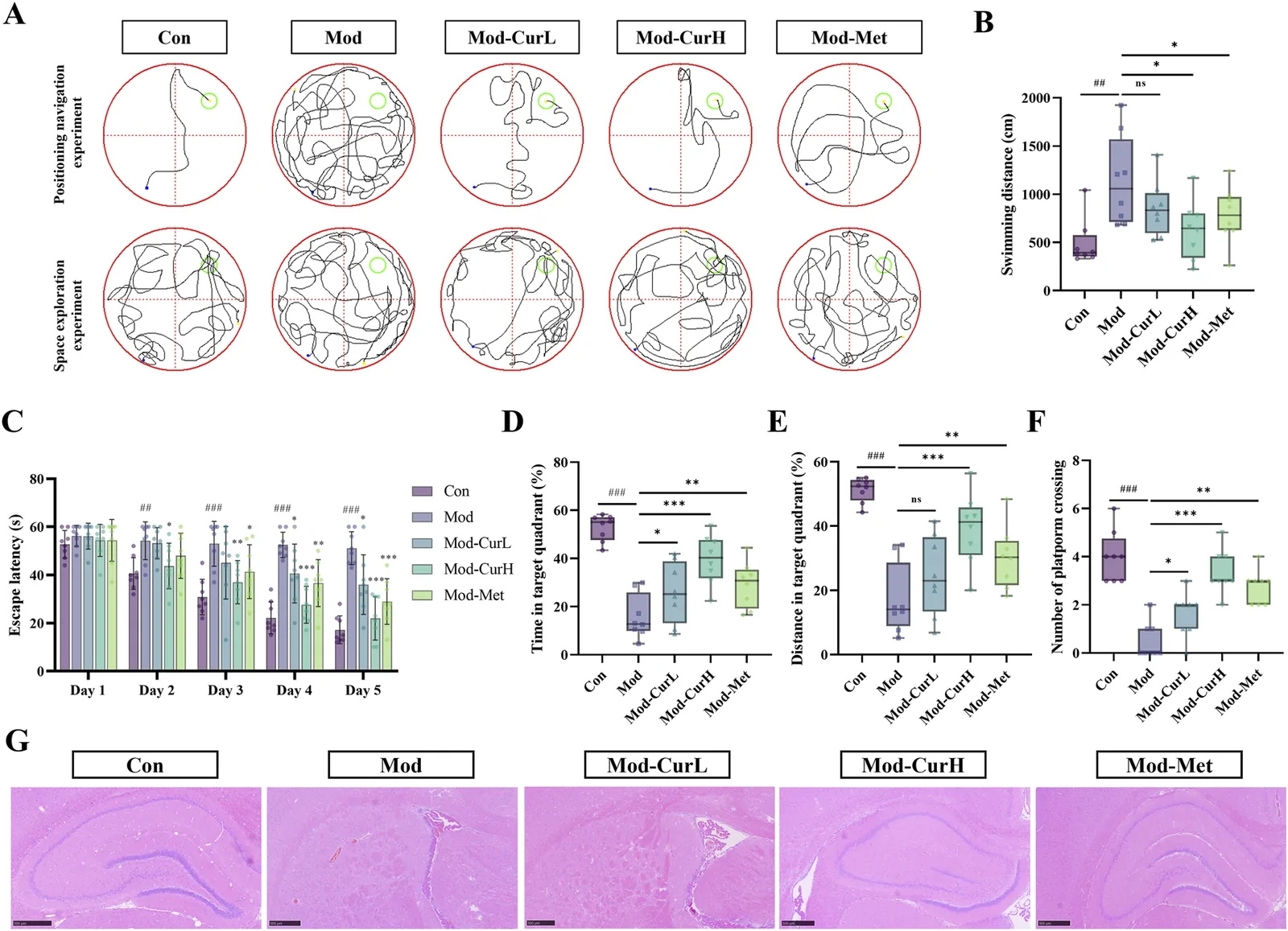
Cur improved cognitive impairment and hippocampal histopathological injury in diabetic rats. **(A)** Representative swimming trajectories in the probe trial of the Morris water maze. **(B,C)** Escape latency and swimming distance during the acquisition phase. **(D–F)** Time spent in the target quadrant, swimming distance in the target quadrant, and platform crossing number in the probe trial. **(G)** Representative H&E staining images of hippocampal tissue from each group. Scale bar = 500 μm. Data are presented as mean ± SD (n = 6–8). #P < 0.05, ##P < 0.01 vs. Con group; *P < 0.05, **P < 0.01 vs. Mod group.

### 3.3 Cur reshaped the brain metabolic profile and attenuated ferroptosis-associated cerebral microvascular injury in diabetic rats

Non-targeted metabolomics was performed to characterise the metabolic alterations associated with Cur treatment. PCA showed clear separation among the Con, Mod, and Cur-treated groups, indicating marked metabolic disturbance in diabetic rats and a shift of the metabolic profile towards that of the Con group after Cur treatment (Figure 3A). Four-quadrant scatter plot analysis identified metabolites that changed in opposite directions in the Mod vs. Con and Cur-treated vs. Mod comparisons (Figure 3B), and the heatmap of the top 50 reversed metabolites showed a similar pattern (Figure 3C). KEGG analysis indicated that these metabolites were mainly associated with ferroptosis, bile secretion, cholesterol metabolism, glycerophospholipid metabolism, primary bile acid biosynthesis, and autophagy (Figure 3D). Further analysis of representative ferroptosis-related metabolites, including glutathione, taurine, arachidonic acid, and adrenic acid, showed significant bidirectional changes, providing additional support for ferroptosis-related metabolic alterations in DE (Supplementary Figure S1). Given the potential involvement of ferroptosis in DE, ferroptosis-associated biochemical and molecular changes were subsequently examined. Brain MDA levels were increased and GSH content was decreased in the Mod group, whereas Cur treatment alleviated these changes (Figures 3E,F). In cerebral microvessel-enriched fractions, ZO-1, Occludin, and Claudin-5 expression was markedly reduced in the Mod group and restored after Cur treatment (Figures 3G,J–L). Meanwhile, ACSL4 expression was increased and GPX4 expression was decreased at both the mRNA and protein levels in diabetic rats, and these alterations were attenuated by Cur treatment (Figures 3G–I,M,N). Collectively, these findings indicate that Cur attenuated ferroptosis-associated metabolic disturbances, oxidative stress, and cerebral microvascular injury in diabetic rats.

**FIGURE 3:**
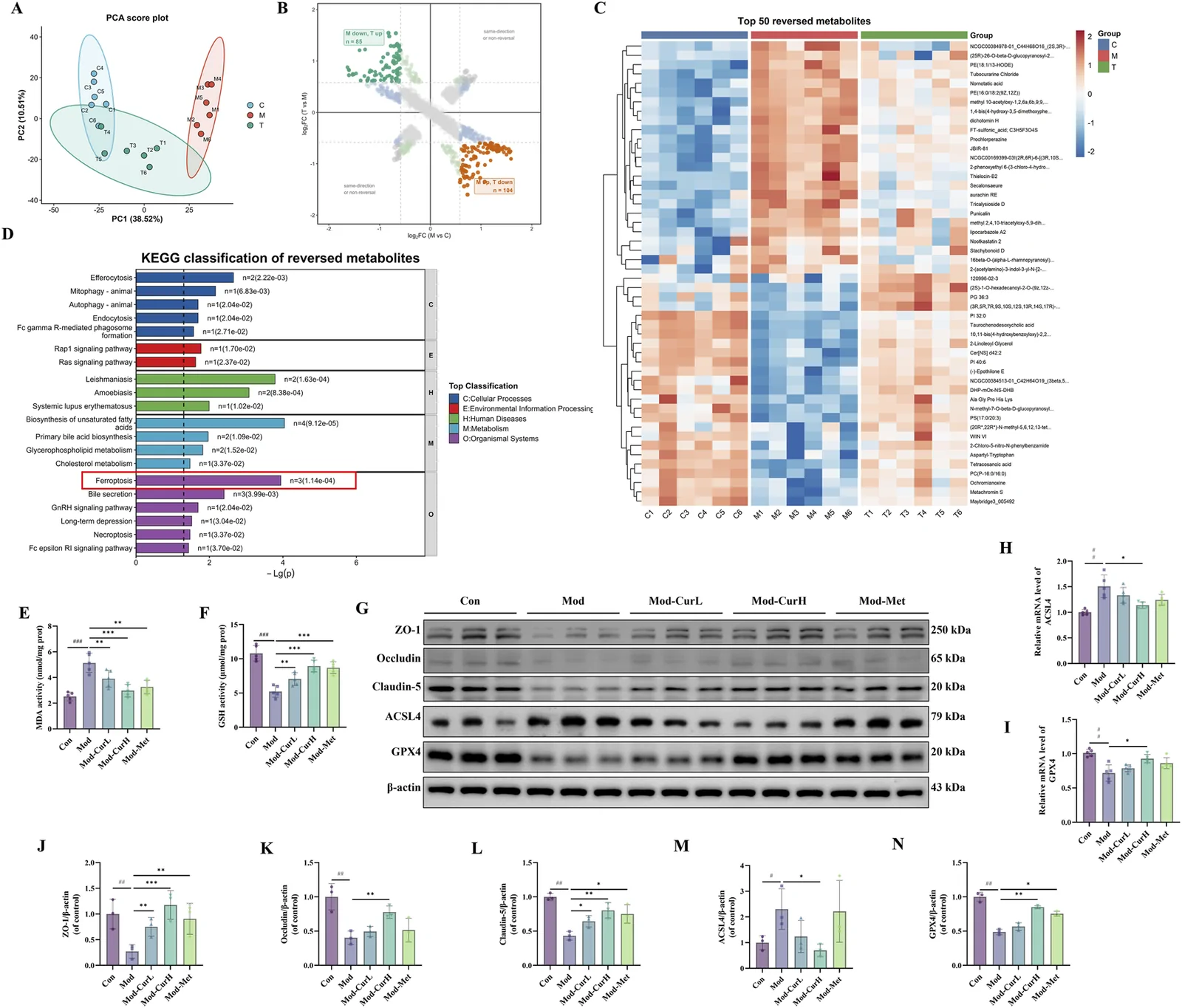
Cur reshaped the brain metabolic profile and attenuated ferroptosis-associated cerebral microvascular injury in diabetic rats. **(A)** PCA score plot. **(B)** Four-quadrant scatter plot of reversed metabolites based on log_2_FC values. **(C)** Heatmap of the top 50 reversed metabolites. **(D)** KEGG classification analysis of reversed metabolites. **(E,F)** MDA and GSH levels in brain tissue. **(G)** Representative western blot bands of ZO-1, Occludin, Claudin-5, ACSL4 and GPX4 in isolated cerebral microvessel-enriched fractions. **(H,I)** Relative mRNA expression levels of ACSL4 and GPX4 in isolated cerebral microvessel-enriched fractions. **(J–N)** Quantitative analysis of ZO-1, Occludin, Claudin-5, ACSL4 and GPX4 protein expression. Data are presented as mean ± SD (n = 3–6). #P < 0.05, ##P < 0.01 vs. Con group; *P < 0.05, **P < 0.01 vs. Mod group.

### 3.4 Network analysis generated candidate hypotheses for experimental evaluation

Network analysis identified 12 overlapping predicted or annotated proteins among the Cur-associated, DE-related, and ferroptosis-related datasets, including BECN1, HIF1A, GCLC, HMOX1, PVT1, GPX4, KEAP1, NFE2L2, CYBB, TP53, NOX4, and ATF4 (Figures 4A–C). Among these proteins, KEAP1, NFE2L2, HMOX1, GPX4, TP53, and NOX4 showed relatively high connectivity within the constructed interaction network. GO enrichment analysis indicated associations with responses to oxidative stress, oxygen levels, and reactive oxygen species metabolism. KEGG analysis included ferroptosis, glutathione metabolism, HIF-1 signalling, reactive oxygen species-related pathways, and neurodegeneration-associated pathways (Figures 4D,E). These network outputs were treated solely as hypothesis-generating predictions and were not interpreted as evidence of pharmacological activity or direct target engagement.

**FIGURE 4:**
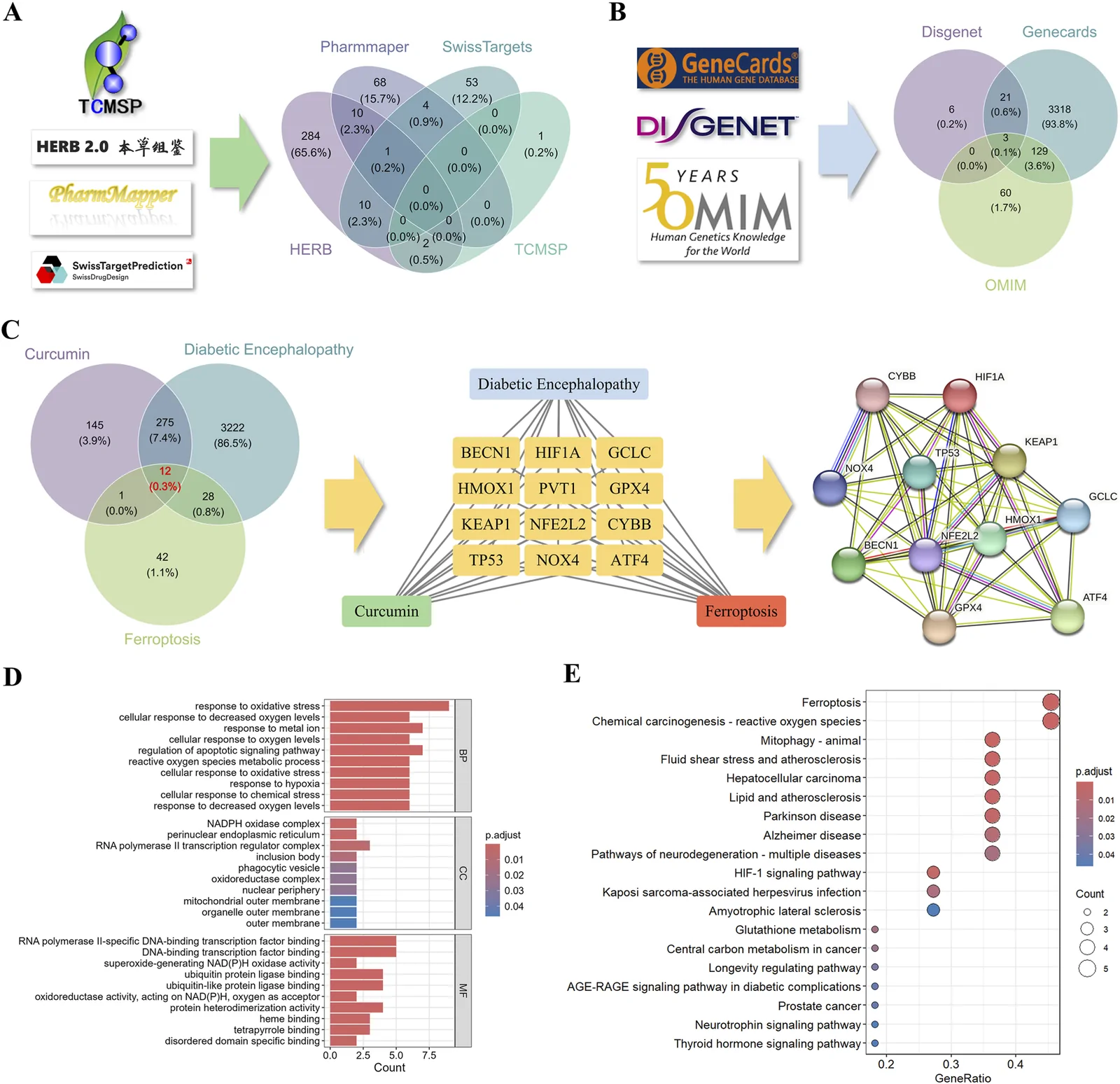
Network analysis of predicted Cur-associated proteins related to diabetic encephalopathy and ferroptosis. **(A)** Workflow of exploratory network analysis. **(B)** Venn diagram showing the intersection of Cur-related targets, DE-related targets, and ferroptosis-related targets. **(C)** Network representation of the overlapping proteins and protein–protein interactions. **(D)** GO enrichment analysis of the overlapping proteins. **(E)** KEGG pathway enrichment analysis of the overlapping proteins.

### 3.5 Cur modulated KEAP1/Nrf2-related signalling in cerebral microvessels of diabetic rats

Consistent with the ferroptosis-related metabolic and biochemical alterations observed *in vivo*, isolated cerebral microvessels from the Mod group exhibited increased KEAP1 and NOX4 mRNA levels and decreased NFE2L2, HMOX1, and GCLC mRNA levels compared with the Con group (Figures 5A–E). Similar changes were observed at the protein level, with increased KEAP1 and NOX4 expression and decreased NRF2, HO-1, and GCLC expression (Figures 5F–K). Cur treatment attenuated these alterations, with CurH generally showing a greater effect, whereas Met induced changes in the same direction. These findings support the involvement of KEAP1/Nrf2-related antioxidant signalling in the protective effects of Cur on cerebral microvessels.

**FIGURE 5:**
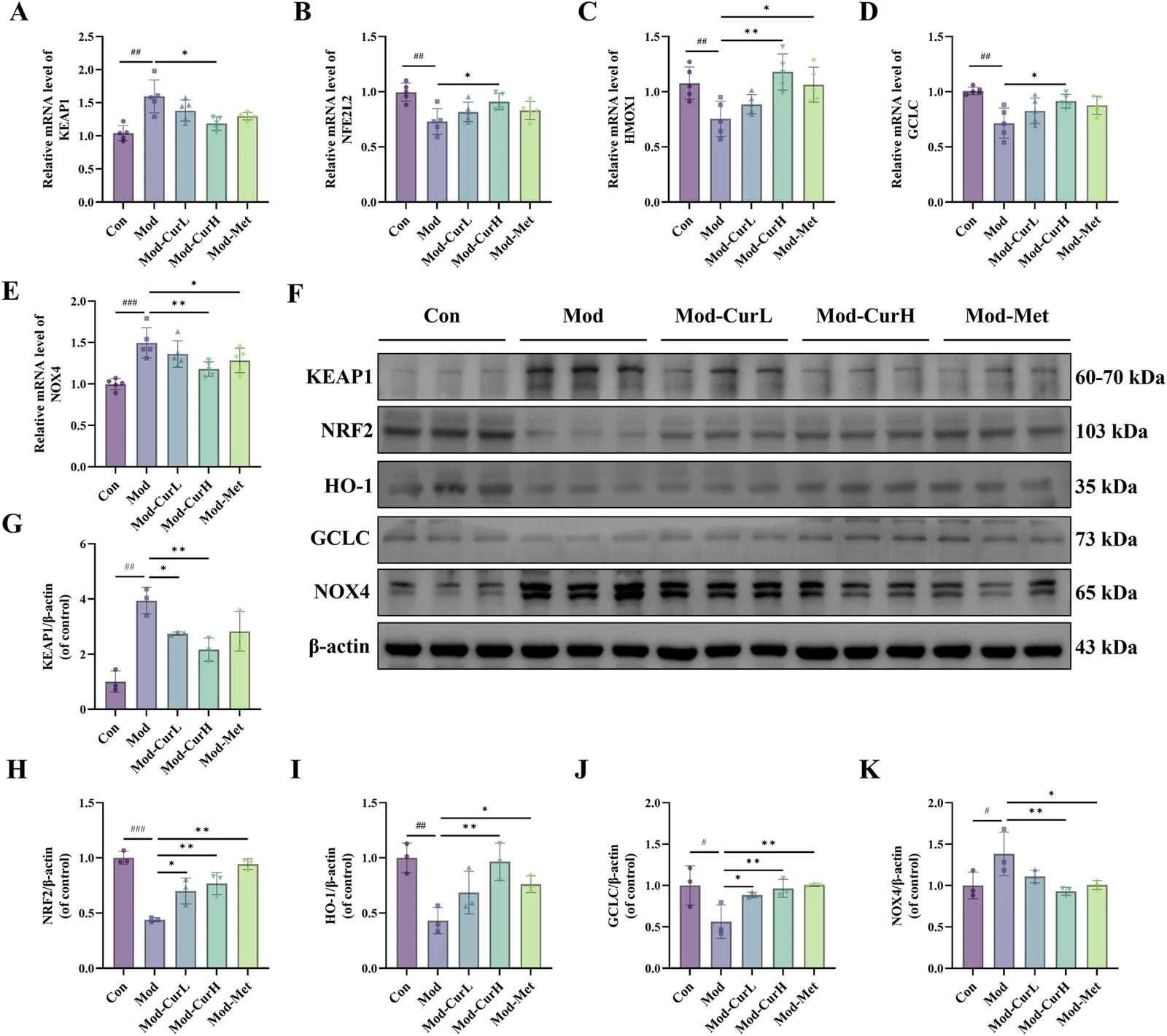
Cur modulated KEAP1/Nrf2-related signalling in cerebral microvessels of diabetic rats. **(A–E)** Relative mRNA expression levels of KEAP1, NFE2L2, HMOX1, GCLC, and NOX4 in isolated cerebral microvessels. **(F)** Representative Western blot bands of KEAP1, NRF2, HO-1, GCLC, and NOX4. **(G–K)** Quantitative analysis of KEAP1, NRF2, HO-1, GCLC, and NOX4 protein expression. Data are presented as mean ± SD, n = 3–5. #P < 0.05, ##P < 0.01 vs. Con group; *P < 0.05, **P < 0.01 vs. Mod group.

### 3.6 HGHF induced ferroptosis-related injury and tight-junction loss in bEnd.3 cells

Based on the CCK-8 results, 25 mM glucose plus 400 μM PA for 24 h was used to establish the *in vitro* HGHF injury model (Figure 6A). Under this condition, intracellular Fe^2+^ levels were increased in the HGHF group, were further elevated by Erastin, and were reduced by Fer-1 (Figure 6B). Tight junction protein expression was also markedly affected, as ZO-1, Occludin, and Claudin-5 were all reduced after HGHF exposure; these changes were aggravated by Erastin and partly reversed by Fer-1 (Figures 6C–F). At both the mRNA and protein levels, ACSL4 was increased whereas GPX4 was decreased in the HGHF group, and these alterations were strengthened by Erastin but attenuated by Fer-1 (Figures 6G–K). Cur showed no obvious cytotoxicity at lower concentrations, whereas 400 μM reduced cell viability (Figure 6L). Based on the CCK-8 screening results, 10 and 20 μM were selected for subsequent experiments (Figure 6M).

**FIGURE 6:**
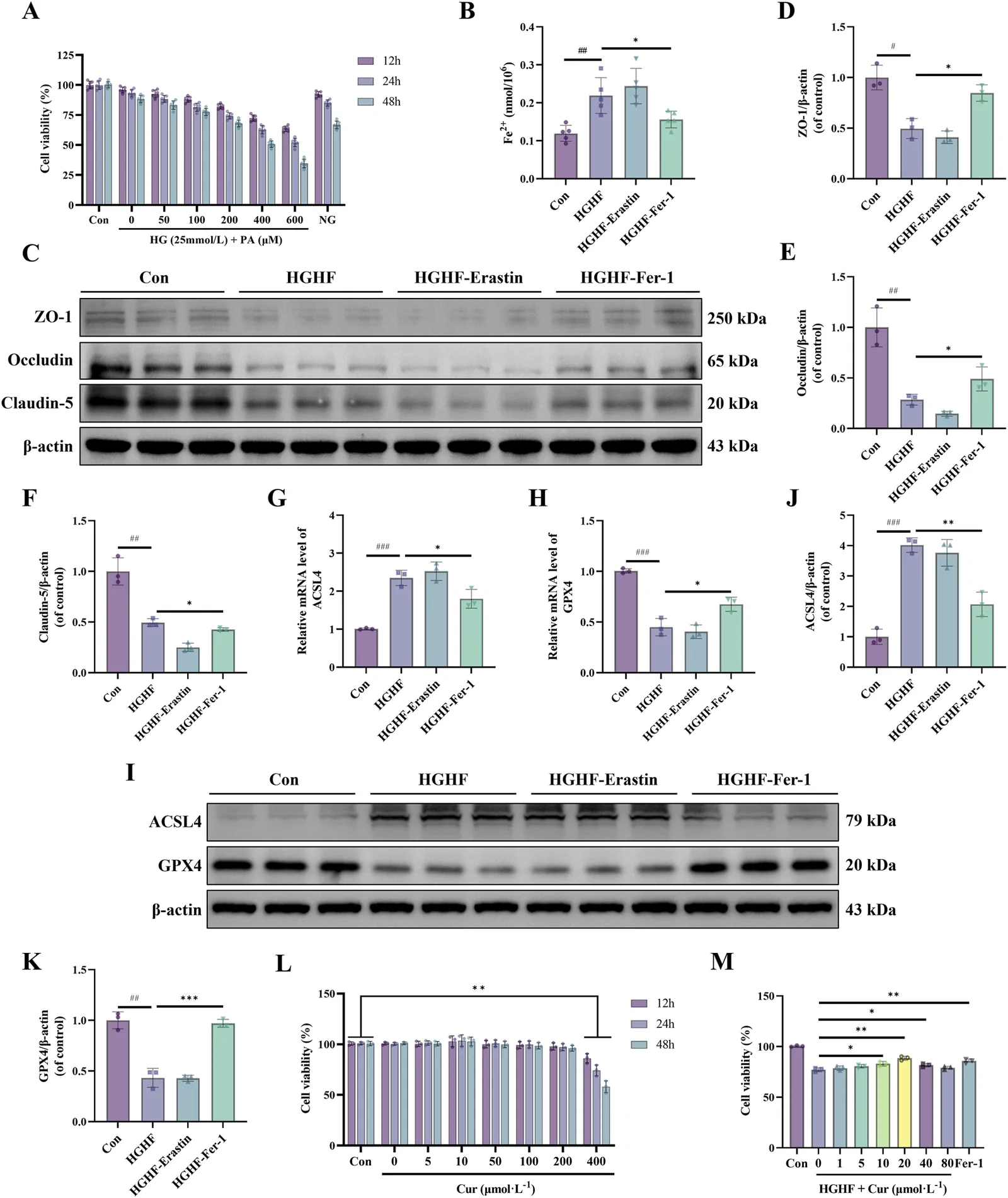
HGHF induced ferroptosis-related injury and tight junction loss in bEnd.3 cells. **(A)** CCK-8 assay used to determine the optimal HGHF injury condition in bEnd.3 cells. **(B)** Intracellular Fe^2+^ levels in each group. **(C)** Representative western blot bands of ZO-1, Occludin, and Claudin-5. **(D–F)** Quantitative analysis of ZO-1, Occludin, and Claudin-5 protein expression. **(G,H)** Relative mRNA expression levels of ACSL4 and GPX4. **(I)** Representative western blot bands of ACSL4 and GPX4. **(J,K)** Quantitative analysis of ACSL4 and GPX4 protein expression. **(L)** CCK-8 assay showing the effect of Cur alone on bEnd.3 cell viability. **(M)** CCK-8 assay used to determine the appropriate Cur concentrations for subsequent experiments. Data are presented as mean ± SD, n = 3–6. #P < 0.05, ##P < 0.01 vs. control group; *P < 0.05, **P < 0.01 vs. HGHF group.

### 3.7 Cur attenuated lipid peroxidation, tight-junction loss, and mitochondrial injury in HGHF-treated bEnd.3 cells

Intracellular ROS accumulation was markedly increased in the HGHF group, whereas both Cur and Fer-1 reduced intracellular ROS levels (Figure 7A). IF staining further showed that ZO-1, Occludin, and Claudin-5 expression was reduced after HGHF exposure and was restored to different extents by Cur and Fer-1 (Figures 7B–E). Consistently, western blot analysis showed decreased ZO-1, Occludin, and Claudin-5 protein expression in the HGHF group, and these changes were improved by Cur treatment, particularly CurH, as well as by Fer-1 (Figures 7F–I). Transmission electron microscopy further revealed typical mitochondrial ultrastructural injury in HGHF-treated bEnd.3 cells, including mitochondrial shrinkage and reduced cristae, whereas these abnormalities were alleviated by Cur treatment (Figure 8A). In parallel, HGHF decreased OPA1, MFN1, and MFN2 expression and increased DRP1 expression, while Cur and Fer-1 partly corrected these changes (Figures 8B–F).

**FIGURE 7:**
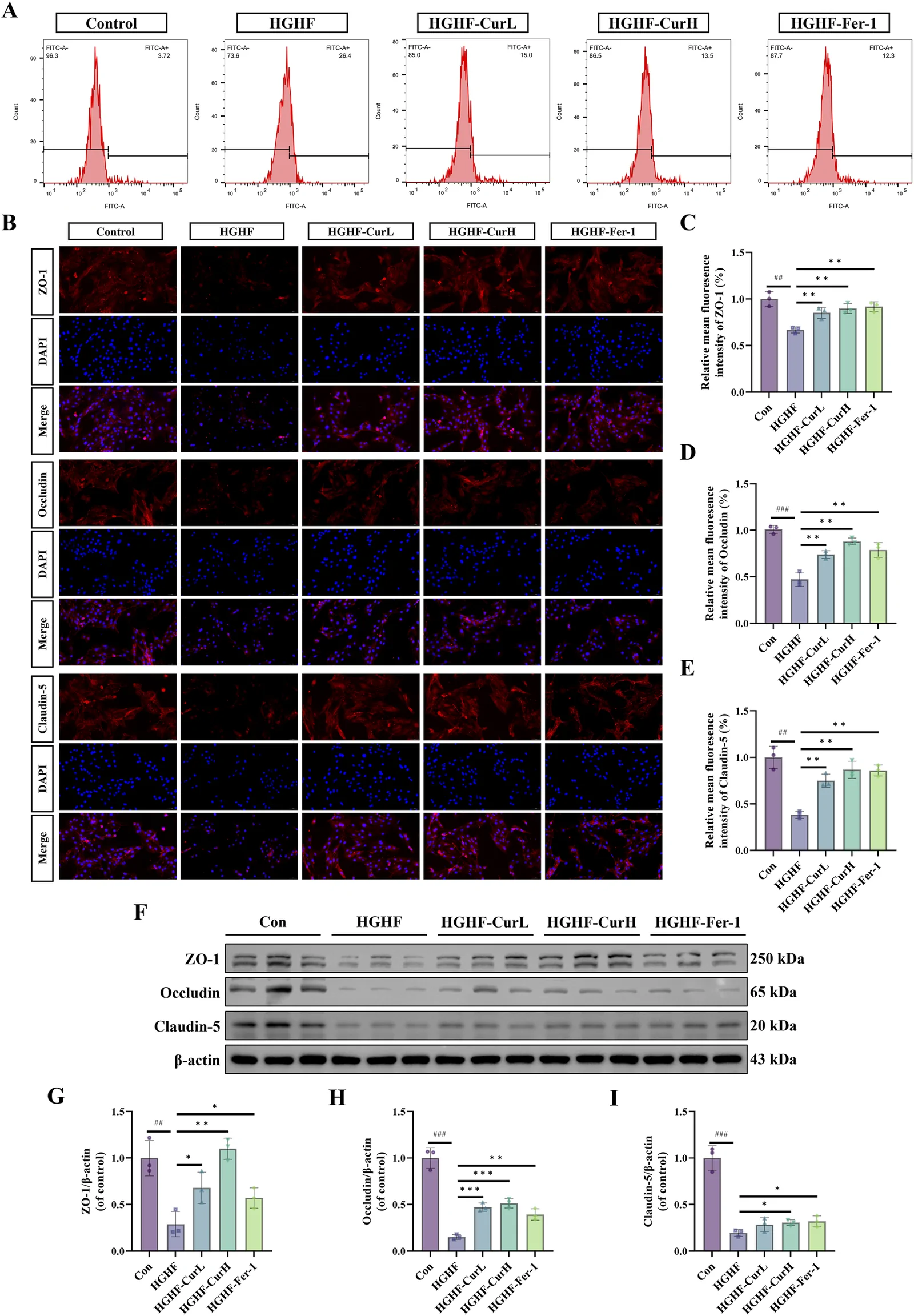
Cur reduced intracellular ROS accumulation and restored tight-junction protein expression in HGHF-treated bEnd.3 cells. **(A)** Flow cytometric analysis of intracellular ROS levels in bEnd.3 cells. **(B)** Representative immunofluorescence images of ZO-1, Occludin, and Claudin-5 expression in each group. **(C–E)** Quantitative analysis of ZO-1, Occludin, and Claudin-5 fluorescence intensity. **(F)** Representative western blot bands of ZO-1, Occludin, and Claudin-5. **(G–I)** Quantitative analysis of ZO-1, Occludin, and Claudin-5 protein expression. Data are presented as mean ± SD, n = 3. #P < 0.05, ##P < 0.01 vs. control group; *P < 0.05, **P < 0.01 vs. HGHF group.

**FIGURE 8:**
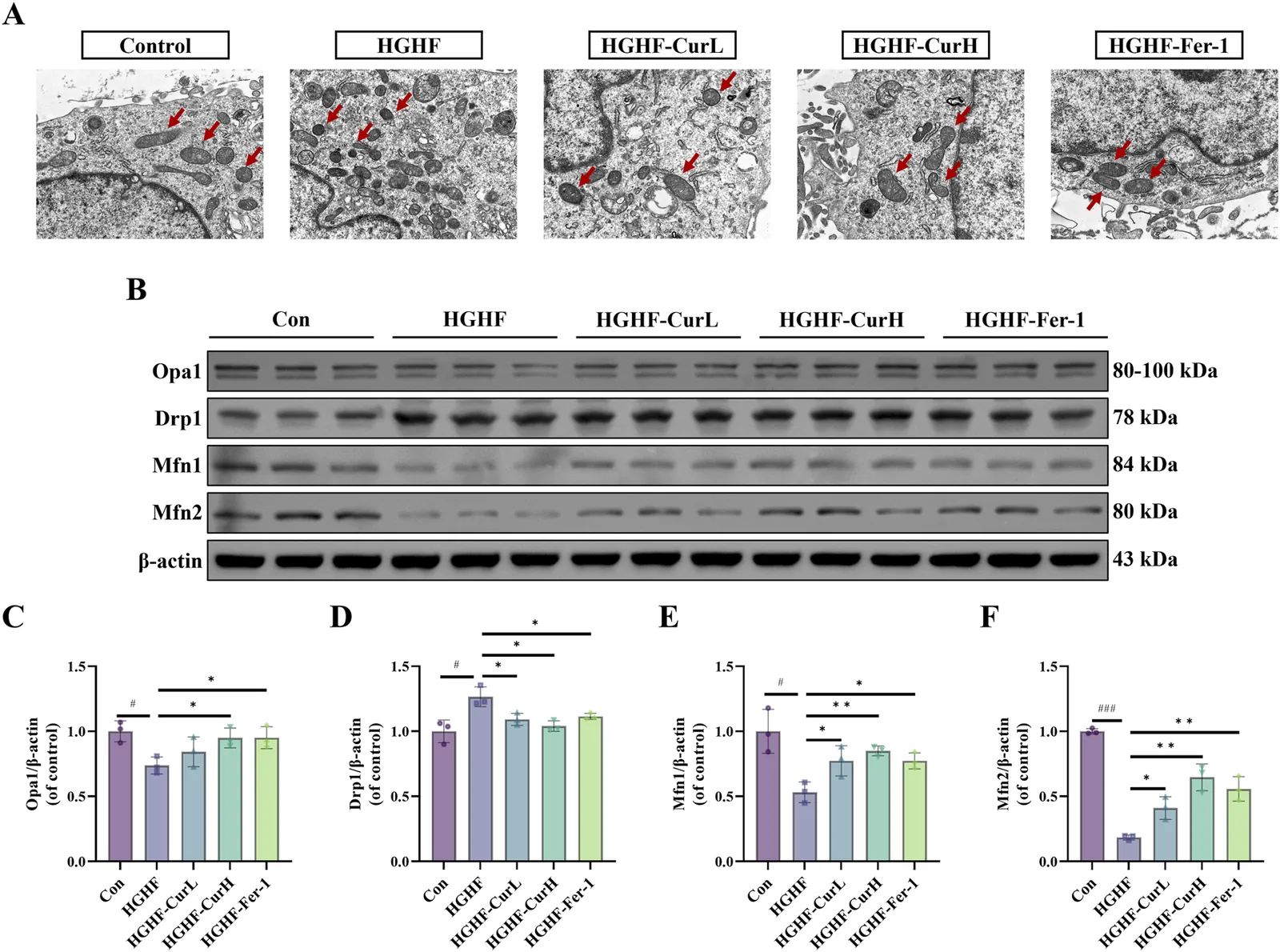
Cur attenuated mitochondrial ultrastructural injury and restored mitochondrial dynamics-related protein expression in HGHF-treated bEnd.3 cells. **(A)** Representative transmission electron microscopy images of mitochondrial ultrastructure in each group. Arrows indicate damaged mitochondria. **(B)** Representative western blot bands of OPA1, MFN1, MFN2, and DRP1. **(C–F)** Quantitative analysis of OPA1, MFN1, MFN2, and DRP1 protein expression. Data are presented as mean ± SD, n = 3. #P < 0.05, ##P < 0.01 vs. Con group; *P < 0.05, **P < 0.01 vs. HGHF group.

### 3.8 Cur improved antioxidant defence and modulated KEAP1/Nrf2-related signalling in HGHF-treated bEnd.3 cells

Compared with the Con group, the HGHF group exhibited decreased GSH levels and increased MDA and Fe^2+^ levels, and these changes were alleviated to different extents by Cur and Fer-1 (Figures 9A–C). At the transcriptional level, KEAP1 mRNA was increased whereas NFE2L2 mRNA was decreased in the HGHF group, and both were partially restored by Cur treatment (Figures 9E,F). At the protein level, KEAP1 and NOX4 expression was elevated, whereas NRF2, HO-1, GCLC, and GPX4 expression was reduced in the HGHF group (Figures 9D,G–L). Cur treatment reversed these changes, with CurH generally showing the stronger effect than CurL, while Fer-1 produced changes in the same direction.

**FIGURE 9:**
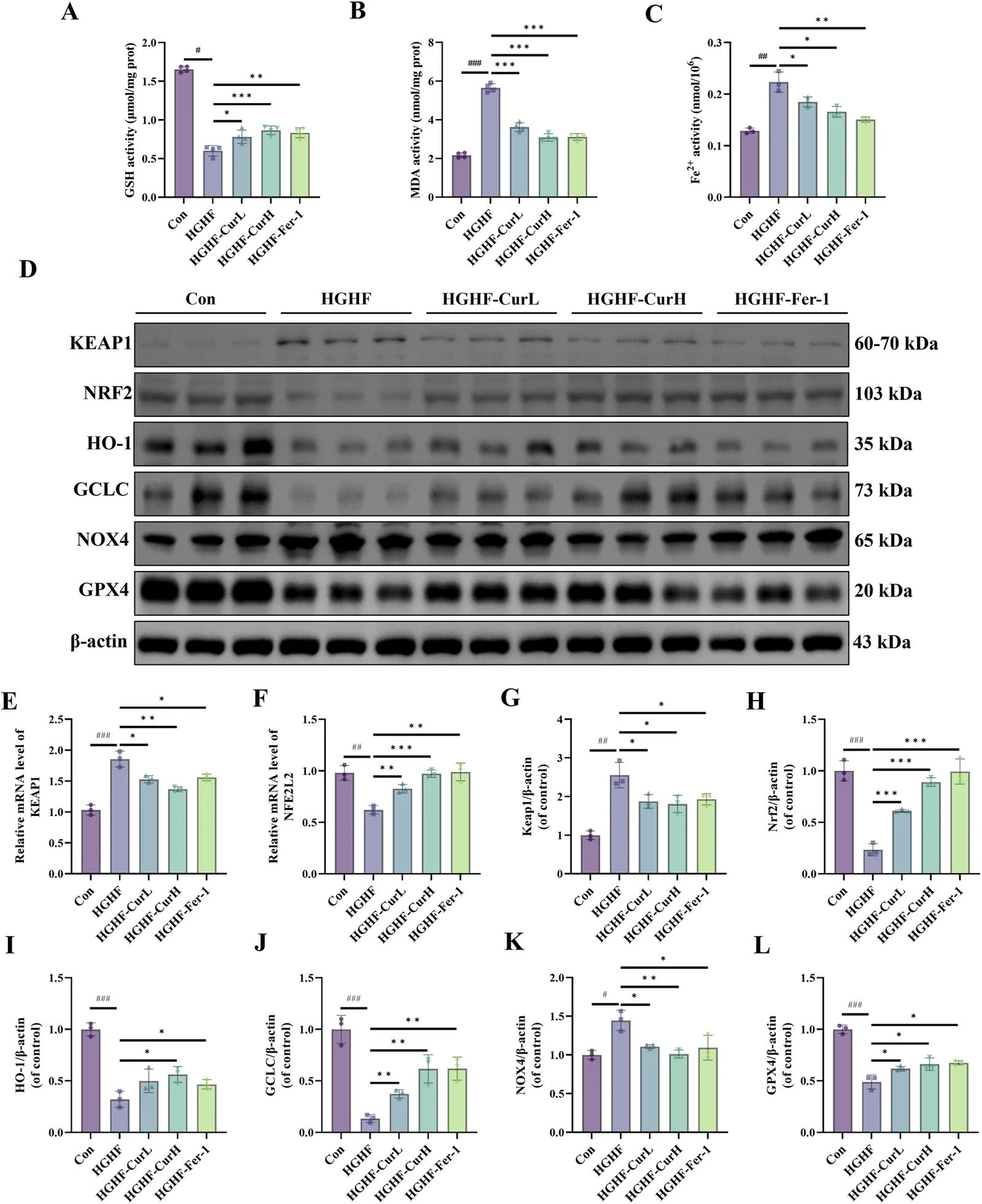
Cur improved antioxidant defence and modulated KEAP1/Nrf2-related signalling in HGHF-treated bEnd.3 cells. **(A–C)** GSH, MDA, and Fe^2+^ levels in each group. **(D)** Representative western blot bands of KEAP1, NOX4, NRF2, HO-1, GCLC, and GPX4. **(E,F)** Relative mRNA expression levels of KEAP1 and NFE2L2. **(G–L)** Quantitative analysis of KEAP1, NOX4, NRF2, HO-1, GCLC, and GPX4 protein expression. Data are presented as mean ± SD, n = 3–4. #P < 0.05, ##P < 0.01 vs. Con group; *P < 0.05, **P < 0.01 vs. HGHF group.

### 3.9 ML385 partially attenuated Cur-mediated protection and KEAP1/Nrf2-related signalling in HGHF-treated bEnd.3 cells

Compared with the HGHF group, CurH increased the expression of ZO-1, Occludin, and Claudin-5, whereas co-treatment with ML385 partially attenuated these effects. ML385 alone further reduced tight-junction protein expression under HGHF conditions (Figures 10A–D). CurH also increased cell viability and GSH levels and decreased MDA and Fe^2+^ levels, while these effects were partially diminished by ML385 (Figures 10E–H). At the transcriptional level, CurH decreased KEAP1 expression and increased NFE2L2 expression. ML385 mainly attenuated the increase in NFE2L2, with a relatively limited effect on KEAP1 expression (Figures 11A,B). At the protein level, CurH decreased KEAP1, ACSL4, and NOX4 expression and increased NRF2, HO-1, GCLC, and GPX4 expression. These changes were partially counteracted by ML385 (Figures 11C–J).

**FIGURE 10:**
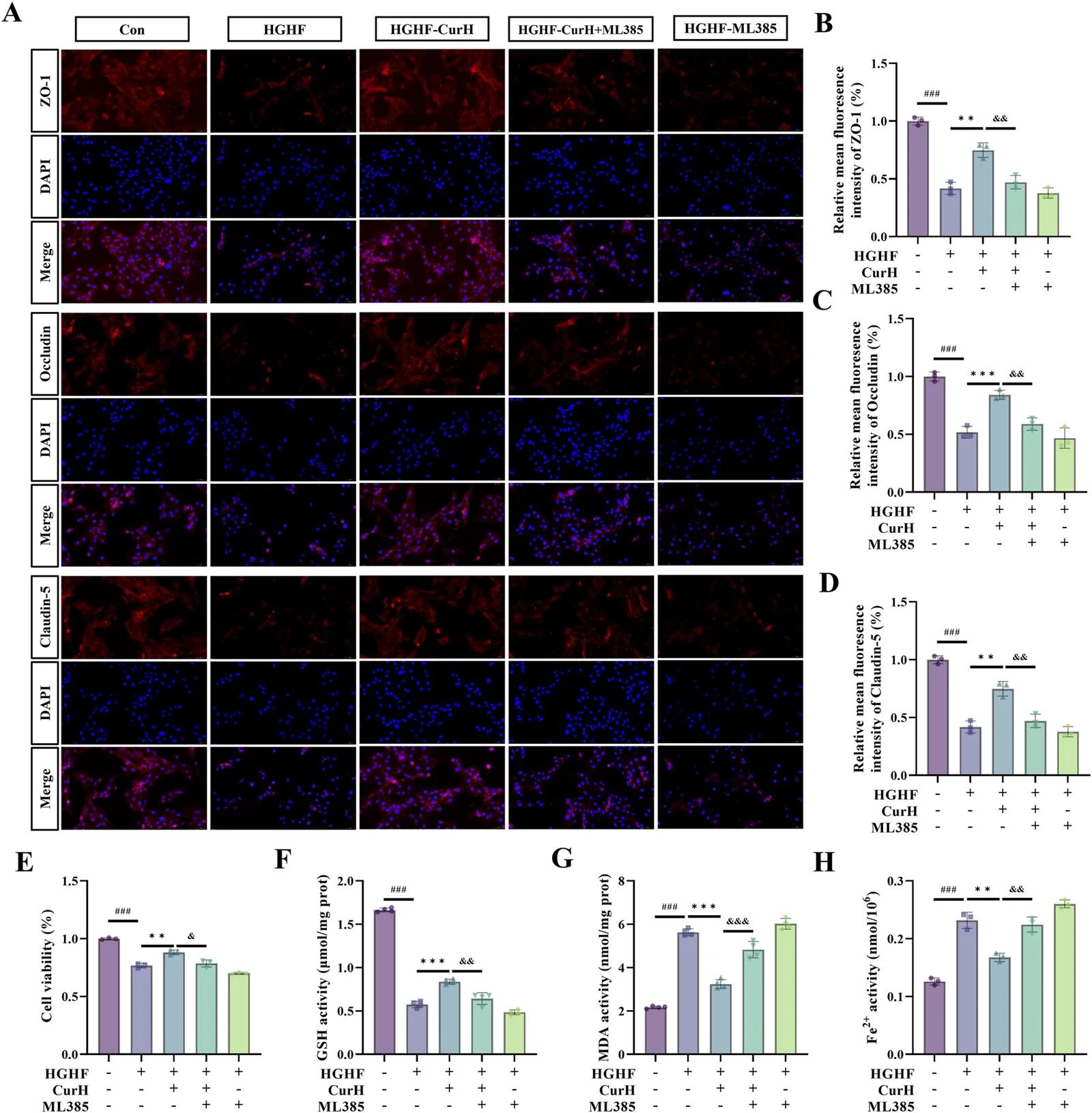
ML385 partially attenuated the protective effects of Cur against HGHF-induced endothelial injury in bEnd.3 cells. **(A)** Representative IF images of ZO-1, Occludin, and Claudin-5 expression in each group. **(B–D)** Quantitative analysis of ZO-1, Occludin, and Claudin-5 fluorescence intensity. **(E–H)** Cell viability, GSH, MDA, and Fe^2+^ levels in each group. Data are presented as mean ± SD, n = 3–4. #P < 0.05, ##P < 0.01 vs. Con group; *P < 0.05, **P < 0.01 vs. HGHF group; &P < 0.05, &&P < 0.01 vs. HGHF + CurH group.

**FIGURE 11:**
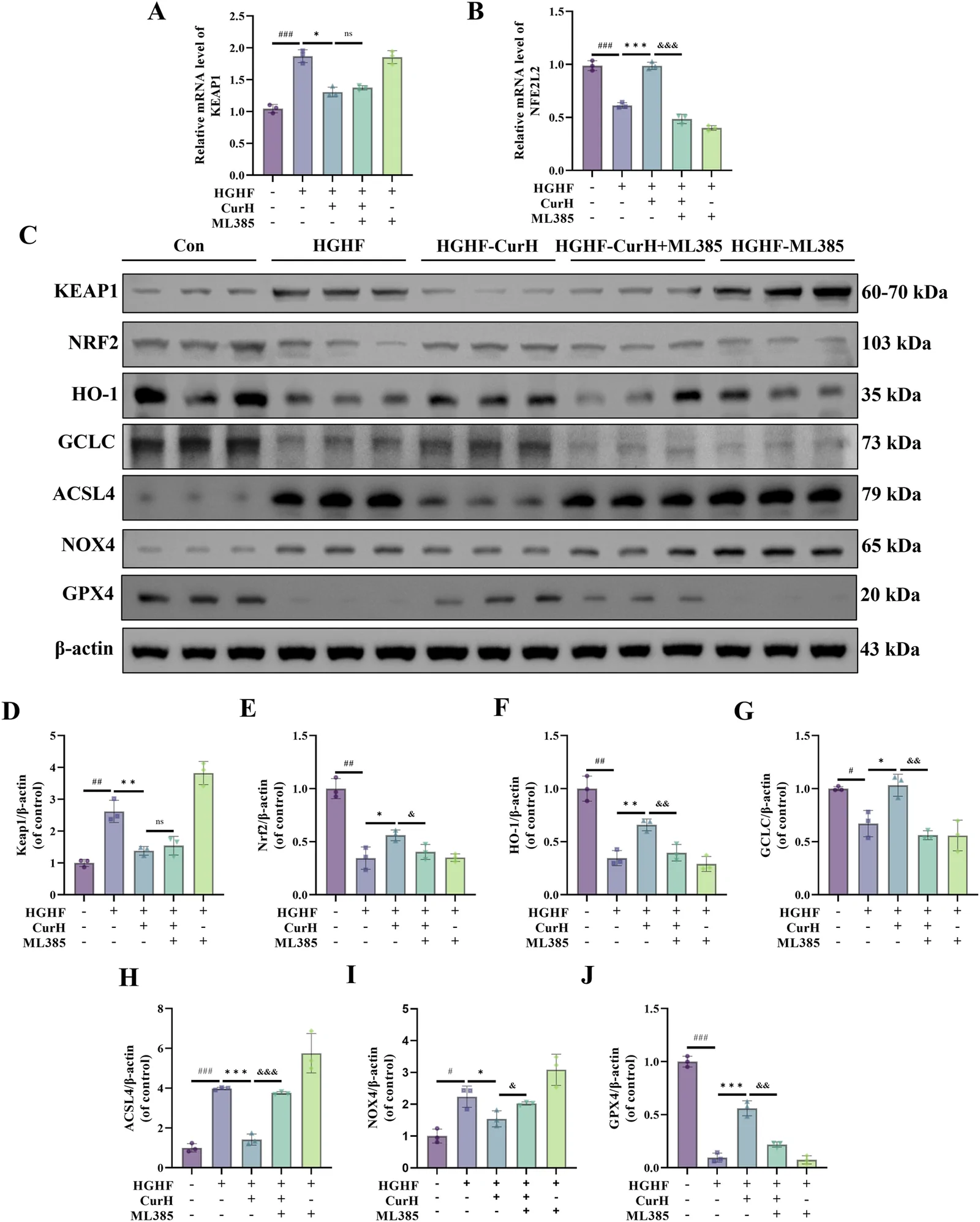
ML385 partially attenuated Cur-mediated regulation of KEAP1/Nrf2-related signalling in HGHF-treated bEnd.3 cells. **(A,B)** Relative mRNA expression levels of KEAP1 and NFE2L2 in each group. **(C)** Representative western blot bands of KEAP1, ACSL4, NOX4, NRF2, HO-1, GCLC, and GPX4. **(D–J)** Quantitative analysis of KEAP1, ACSL4, NOX4, NRF2, HO-1, GCLC, and GPX4 protein expression. Data are presented as mean ± SD, n = 3. #P < 0.05, ##P < 0.01 vs. Con group; *P < 0.05, **P < 0.01 vs. HGHF group; &P < 0.05, &&P < 0.01 vs. HGHF + CurH group.

## 4 Discussion

The present study showed that Cur ameliorated metabolic disturbances, cognitive impairment, hippocampal injury, and cerebral microvascular abnormalities in experimental DE. Cur also attenuated ferroptosis-associated endothelial injury under HGHF conditions. By integrating brain metabolomics, isolated cerebral microvessel analysis, endothelial cell experiments, ferroptosis-modifying pharmacological interventions, and Nrf2 inhibition, our findings further implicate cerebral microvascular ferroptosis and KEAP1/Nrf2-related signalling in the protective effects of Cur. Collectively, these results support a neurovascular mechanism in which Cur alleviates DE-associated injury by improving systemic metabolic homeostasis and limiting oxidative and ferroptosis-related damage in the cerebral microvasculature.

DE is increasingly understood as a chronic brain complication arising from the convergence of systemic metabolic dysregulation and progressive intracerebral injury rather than as a simple secondary consequence of hyperglycaemia alone (Huai et al., 2025). Oxidative stress, neuroinflammation, pyroptosis, and maladaptive metabolic reprogramming interact throughout disease progression and collectively contribute to neuronal and vascular dysfunction (Luo et al., 2024; Nagayach et al., 2024; Zhuang et al., 2025). Within this multifactorial context, experimental studies in diabetic rats have shown that Cur attenuates cerebral and hippocampal oxidative stress, inflammatory responses, and histopathological injury while improving endogenous antioxidant defences, including GSH, SOD, and catalase (Faheem and El Askary, 2017; Miao et al., 2021; Salgintaş et al., 2021; Özsan et al., 2024). These findings are consistent with our observations that Cur improved glucose and lipid homeostasis, cognitive performance, and hippocampal morphology and reduced oxidative stress in diabetic rats.

An important feature of the present work is the emphasis on cerebral microvascular injury. Increasing evidence suggests that diabetes-associated cognitive decline should be interpreted not only from a neuron-centred perspective but also within a neurovascular-unit framework, in which cerebral microvascular dysfunction contributes actively to brain vulnerability (Bogush et al., 2017; van Sloten et al., 2020; Wątroba et al., 2023). Diabetes-associated blood–brain barrier injury is accompanied by increased endothelial permeability and reduced expression of tight-junction proteins, including ZO-1, Occludin, and Claudin-5 (Zhao et al., 2019; Yin et al., 2024). Cur has also been reported to preserve brain endothelial barrier function and tight-junction protein expression under injurious conditions (Wang et al., 2013). Moreover, disruption of the blood–brain barrier can amplify neuroinflammatory responses and cognitive deterioration (Geng et al., 2018). Against this background, our findings in isolated cerebral microvessels and bEnd.3 cells support cerebral microvascular injury as a biologically relevant component of experimental DE and suggest that preservation of endothelial tight-junction-associated proteins contributes to the protective effects of Cur.

The present data further support ferroptosis as an important pathological process associated with diabetic cerebral microvascular injury. Ferroptosis is increasingly implicated in blood–brain barrier disruption, and brain microvascular endothelial cells are susceptible to ferroptosis-associated injury under pathological conditions (Fang et al., 2022; Liu et al., 2023; Zhao et al., 2023). More broadly, ferroptosis has been linked to the pathogenesis of diabetes and its complications (Liu et al., 2024). Experimental studies of diabetes-associated cognitive dysfunction have further implicated ferroptosis in hippocampal injury and cognitive decline (Shi et al., 2023; Cheng et al., 2024a; Ma et al., 2025). Consistent with these observations, brain metabolomics in the present study revealed alterations in ferroptosis-related metabolic pathways, while changes in glutathione, taurine, arachidonic acid, and adrenic acid further indicated disturbances in redox and lipid metabolism associated with ferroptosis. GSH is required for GPX4-dependent detoxification of lipid hydroperoxides, whereas arachidonic acid- and adrenic acid-containing phospholipids represent major substrates for ferroptotic lipid peroxidation (Kagan et al., 2017). The increases in MDA, Fe^2+^, and ACSL4, together with decreases in GSH and GPX4, provided additional biochemical and molecular evidence of ferroptosis-associated injury. Importantly, the aggravation of endothelial injury by erastin and its attenuation by Fer-1 further support the involvement of ferroptosis in the endothelial phenotype observed in our experimental models.

Another noteworthy finding is that ferroptosis-associated endothelial injury was accompanied by disturbances in mitochondrial homeostasis. Mitochondrial dysfunction is increasingly recognised as a central feature of diabetic endothelial injury, and disruption of the mitochondrial fission–fusion balance has been associated with endothelial oxidative stress and functional impairment (Shenouda et al., 2011; Fang et al., 2024). This is particularly relevant to the blood–brain barrier, where endothelial mitochondria contribute to energy metabolism, barrier regulation, and vascular resilience (Shao et al., 2025). In the present study, HGHF exposure decreased OPA1, MFN1, and MFN2 expression, increased DRP1 expression, and induced mitochondrial shrinkage and cristae loss, whereas Cur and Fer-1 partly attenuated these abnormalities. These findings suggest that the effects of Cur on ferroptosis-associated endothelial injury extend beyond the suppression of lipid peroxidation and are accompanied by improvement in mitochondrial homeostasis.

At the mechanistic level, the coordinated regulation of KEAP1, NRF2, HO-1, GCLC, GPX4, ACSL4, and NOX4 provides a plausible signalling framework linking the antioxidant and anti-ferroptotic effects of Cur. Nrf2 is an important regulator of cellular redox homeostasis and ferroptotic susceptibility and contributes to blood–brain barrier integrity by regulating antioxidant and cytoprotective responses (Cazalla et al., 2024; Xiang et al., 2024). Activation of Nrf2 has also been shown to attenuate blood–brain barrier injury by restraining oxidative stress, ferroptosis, and inflammation (Fan et al., 2024). Cur can promote Nrf2 stabilisation and activation (Park et al., 2021), and Cur-mediated regulation of Nrf2-related signalling has been associated with reduced oxidative stress and ferroptosis in other diabetic complications (Wei et al., 2022; Wu et al., 2022; Xue et al., 2024). In DE, hyperglycaemia- and AGE-induced oxidative stress has also been linked to impaired mitophagy through KEAP1/Nrf2-related signalling (Xu et al., 2024). In the present study, exploratory network analysis was used only to generate candidate hypotheses, whereas the involvement of KEAP1/Nrf2-related signalling was evaluated experimentally through the observed molecular changes and the effects of ML385. Furthermore, ML385 partially attenuated the effects of Cur on antioxidant defence, ferroptosis-related markers, and tight-junction protein expression. These findings indicate that KEAP1/Nrf2-related signalling contributes to the protective effects of Cur. However, Cur has been recognised as a pan-assay interference compound, and its promiscuous or assay-dependent behaviour requires particular caution when interpreting computational predictions and isolated *in vitro* findings (Nelson et al., 2017; Bolz et al., 2021; Magalhães et al., 2021). Accordingly, the network analysis in this study was considered hypothesis-generating rather than evidence of pharmacological activity or direct target engagement. In addition, overlap among database sources, heterogeneous curation, similarity-based prediction, and unquantified false-positive and false-negative rates further limit the interpretation of the network results. Greater mechanistic weight is therefore placed on the convergence of the *in vivo* findings, cerebral microvessel data, ferroptosis-modifying interventions, and partial reversal by ML385. The incomplete reversal by ML385 further suggests that additional redox, metabolic, inflammatory, or mitochondrial mechanisms may participate in the protective effects of Cur.

This study has several strengths, including the integration of animal experiments, behavioural assessment, hippocampal histopathology, non-targeted metabolomics, cerebral microvessel analysis, endothelial cell modelling, ultrastructural assessment, ferroptosis pharmacology, and pathway inhibition. Nevertheless, several limitations should be acknowledged. First, bEnd.3 cells are an immortalised murine brain endothelial cell line and do not fully reproduce the barrier phenotype and biological characteristics of primary or human brain microvascular endothelial cells, which limits the translational interpretation of the *in vitro* findings (Sun et al., 2022). Second, although ML385 provides pharmacological evidence for the involvement of Nrf2, potential off-target effects and the absence of Nrf2 knockdown or knockout experiments preclude definitive causal attribution. In addition, given the recognised PAINS properties of Cur, potential nonspecific or assay-dependent effects in the cellular experiments cannot be completely excluded; nevertheless, the interpretation of the present study is based on convergent biochemical, molecular, imaging, and pharmacological evidence rather than on a single assay or computational prediction (Nelson et al., 2017; Bolz et al., 2021; Magalhães et al., 2021). Third, Cur was administered at relatively high oral doses of 200 and 400 mg/kg. Although no detectable adverse changes were observed in the measured safety-related parameters in healthy rats, this finding reflects tolerability within the present experimental setting and does not establish pharmacokinetic or translational relevance. Cur is characterised by poor aqueous solubility, limited systemic bioavailability, and extensive metabolism after oral administration, and pharmacokinetic or brain-exposure measurements were not performed in the present study (Sharma et al., 2007; Hao et al., 2023). Thus, these experimental doses should not be interpreted as directly translatable human therapeutic doses, and potential nonspecific effects associated with the higher dose cannot be completely excluded. Similarly, although the 10 and 20 μM concentrations used *in vitro* were selected following concentration–response viability screening, their relationship to physiologically achievable endothelial exposure remains uncertain. Fourth, cerebral microvascular protection was evaluated mainly through changes in ZO-1, Occludin, and Claudin-5 expression and experiments using bEnd.3 cells. Direct functional assessments of blood–brain barrier integrity, such as tracer extravasation *in vivo* or transendothelial electrical resistance and paracellular permeability measurements *in vitro*, were not included. Finally, whole-brain non-targeted metabolomics cannot determine whether the observed metabolic alterations originated specifically from cerebral endothelial cells. As DE involves interactions among endothelial cells, neurons, astrocytes, microglia, and pericytes, the relationship between endothelial ferroptosis and the wider neurovascular unit requires further investigation.

Overall, the present findings support a model in which chronic diabetic metabolic stress promotes cerebral microvascular oxidative injury, tight-junction disruption, mitochondrial dysfunction, and ferroptosis-associated metabolic and molecular alterations, thereby contributing to hippocampal injury and cognitive dysfunction. Cur attenuated these pathological changes *in vivo* and *in vitro*, while the coordinated molecular responses and the partial effect of ML385 indicate that KEAP1/Nrf2-related signalling contributes to this protection together with other redox, metabolic, and mitochondrial mechanisms. These findings extend the current understanding of the neurovascular protective effects of Cur in experimental DE and support further investigation of cerebral microvascular ferroptosis as a potential intervention target.

## 5 Conclusion

Our findings indicate that Cur attenuates DE-related metabolic dysregulation, cognitive impairment, hippocampal injury, and ferroptosis-associated cerebral microvascular injury. These effects were accompanied by improvements in oxidative stress, tight-junction protein expression, and mitochondrial homeostasis, together with modulation of KEAP1/Nrf2-related signalling (Figure 12). By linking cerebral microvascular ferroptosis with hippocampal injury and cognitive dysfunction in diabetes, this study extends the current understanding of the neurovascular mechanisms underlying DE and highlights cerebral microvascular endothelial injury as a relevant component of Cur-mediated protection. These findings support further investigation of Cur and other ferroptosis-modulating strategies for diabetes-associated cognitive impairment. Future studies should clarify the causal contribution of this pathway through endothelial-specific manipulation of KEAP1/Nrf2, examine the interactions between endothelial cells and other components of the neurovascular unit, optimise the pharmacokinetic properties and delivery of Cur, and validate these findings in primary or human-derived endothelial models and additional models of diabetes-associated cognitive impairment.

**FIGURE 12:**
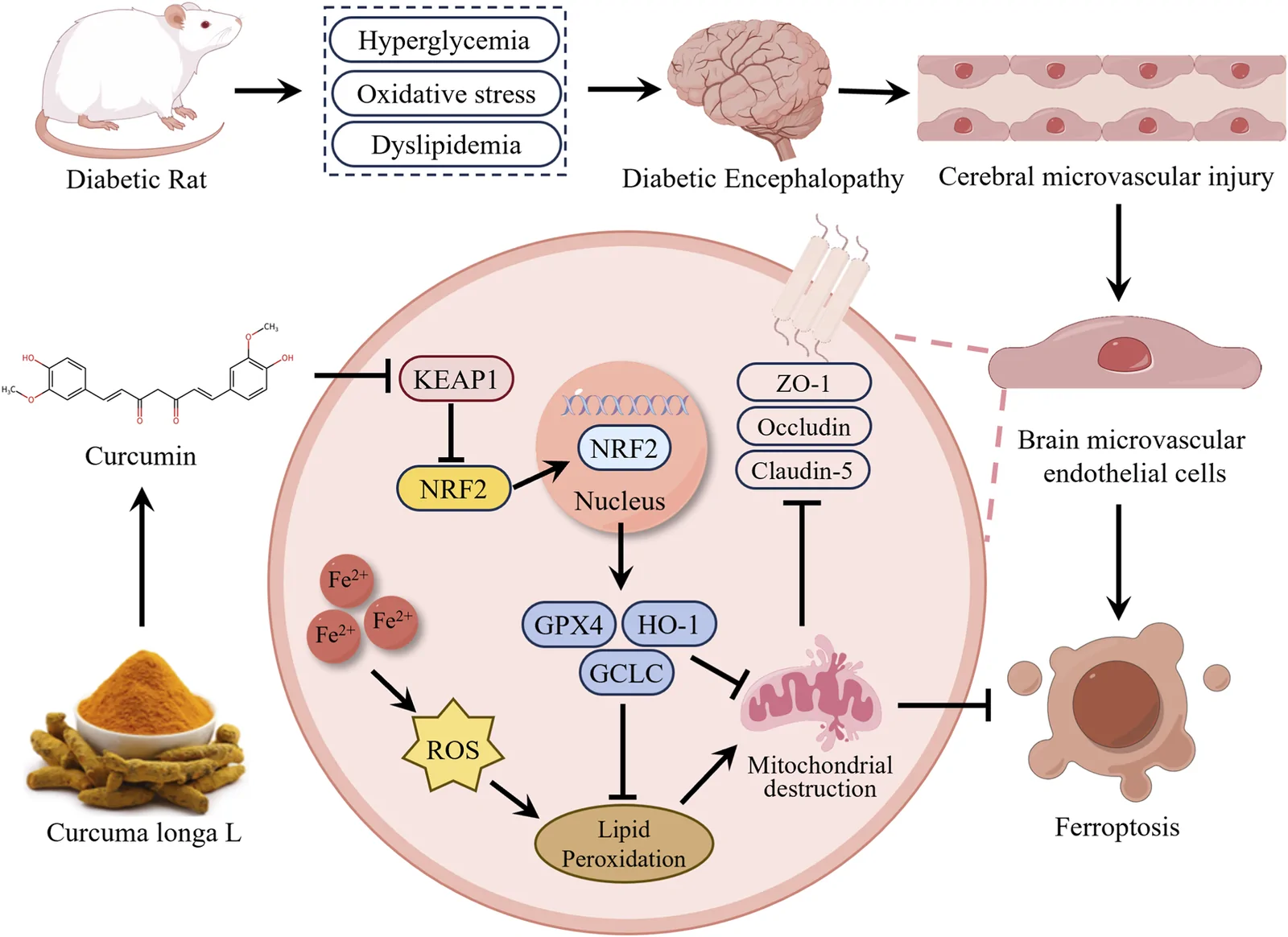
Graphical abstract. Adapted with permission from https://www.figdraw.com/#/. Used in accordance with the applicable Figdraw license and terms of use.

## Data Availability

The original contributions presented in the study are included in the article/Supplementary Material, further inquiries can be directed to the corresponding authors.
