# Supplementary material for: Curcumin alleviates diabetic encephalopathy by attenuating cerebral microvascular ferroptosis: involvement of KEAP1/Nrf2 signalling
Source: Front Pharmacol. 2026 Sep 21;17:1914347. doi: 10.3389/fphar.2026.1914347 (PMC13635145; doi:10.3389/fphar.2026.1914347)
Supplement: Supplementary file 1 [file Supplementaryfile1.docx]

Supplementary Material

# 1 Supplementary Figures

**Supplementary Figure 1.** **Cur partially normalized representative ferroptosis-related metabolic alterations in diabetic rats.** (A) Bidirectional log₂ fold changes of representative ferroptosis-related metabolites, including arachidonic acid, adrenic acid, glutathione, and taurine, in the Mod vs. Con and Cur vs. Mod comparisons. (B–E) Log₂-normalized peak intensities of taurine, arachidonic acid, adrenic acid, and glutathione, respectively. Data are presented as boxplots with individual data points (n = 6). Statistical significance was determined using FDR-adjusted pairwise comparisons. **FDR < 0.01.
